# Exploring the healthcare experiences and support needs of chestfeeding or breastfeeding for trans and non-binary parents based in the United Kingdom

**DOI:** 10.1080/26895269.2023.2265371

**Published:** 2023-10-09

**Authors:** Jessica Eve Jackson, Ray Wild, Jenny Hallam, Rudy Graves, B. J. Woodstein, Penny Stothard

**Affiliations:** a College of Health, Psychology and Social Care, University of Derby, Derby, UK; b Leeds Teaching Hospital NHS Trust, Leeds, UK; c University of East Anglia, Norwich, UK

**Keywords:** Breastfeeding, chestfeeding, child and family health, infant feeding, non-binary health, trans health

## Abstract

***Background:*** Trans and/or non-binary parents experience structural exclusion in family healthcare and there is a need for specialist training for healthcare professionals so that they are able to create a gender inclusive environment.

***Aim:*** As part of a continued effort to address health inequity this study explores the body experiences of infant feeding within trans and non binary communities.

***Method:*** Semi structured interviews were conducted with seven trans or non-binary parents, based in the United Kingdom, who have experienced chestfeeding or breastfeeding.

***Results:*** A reflexive thematic analysis was utlitised to identify three main themes which centered around baby, body and support in health care settings. Parents were informed of the benefits of their milk and were strongly motivated by their child’s needs. However, body feeding was emotionally and physically challenging.

***Conclusion:*** Person-centered care with consistent attention to language is required when supporting lactation.

## Background

Trans and/or non-binary (TGNB) people face multiple social and personal challenges, as well as barriers to healthcare, during childbearing, baby feeding, and early parenting (Botelle et al., 2021; Falck et al., 2021; Winter et al., 2016). As such, TGNB parents, and their babies, require suitably informed and culturally competent healthcare and support while establishing infant feeding and their transition to parenthood (Botelle et al., 2021). Whilst there has recently been growing public, professional and academic awareness of the lactation needs of TGNB people (MacDonald, 2019) the evidence base in this area is limited. To address this issue the current paper uses qualitative methods to explore the lactation experiences of TGNB people within a UK context.

Previous research exploring transmasculine people’s experiences of chest feeding has revealed that issues surrounding gender dysphoria important (MacDonald et al., 2016). For some participants navigating gender-affirming top surgery was a challenge and future bodyfeeding^1^ plans were not shared with surgeons as it was felt that this would jeopardize access to surgery. For some participants, gender dysphoria intensified whilst chestfeeding whereas others experienced this after weaning as their mammary tissue no longer had a practical purpose. Oberhelman-Eaton et al. (2022) further extended this research using a case study approach to understand gender-affirming testosterone in a lactating transgender man. Such findings are important for raising awareness for healthcare professionals (HCPs) in supporting transmasculine people in their infant feeding choices but also for advising trans youth who are considering starting a family in the future. As reported by Lowik et al. (2023) pregnancy and starting a family is seen by trans youth as something that needs to be done before transition. Thus, further advice and support around pregnancy and body feeding could be beneficial to this group.

The evidence base surrounding transgender women is more limited with a focus on single case studies (Reisman & Goldstein, 2018; Wamboldt et al., 2021). These case studies have a medical focus on the use of hormones and the practicalities of inducing lactation. The wider cultural backlash in response to these studies surrounding ‘natural’ and ‘hormone-free’ milk highlighted the importance of addressing misunderstandings and a need for HCPs to further understand the stigma faced by transgender parents (Paynter, 2019). Indeed, it is argued that there is a need for specialist LGB and TGNB training for HCPs and for HCPs to provide cues to patients that they are knowledgeable and gender-inclusive (MacDonald, 2019).

Within healthcare settings, Norris and Borneskog (2022) have suggested that HCPs evidence what they term the ‘Cisnormative Blindspot’.^2^ For example, when starting a parenting journey, TGNB parents are forced into a highly cisheteronormative environment in which they find themselves frequently pragmatically balancing their identities with healthcare assumptions and a lack of knowledge. Within this paradigm, trans parents’ experiences are thought to be ignored, erased, or treated with prurient interest or stigma. This is significant as in England there is recognition that parents who face additional barriers to healthcare may need access to universal services in addition to targeted programmes tailored to specific needs to reduce inequality (Pearce et al., 2019). However, NHS England has only acknowledged trans parents as users of maternity services since 2022 (Crowther et al., 2022). A lack of a gender-affirming healthcare environment can limit access to care and further contribute to the discrimination of marginalized populations (MacLean, 2021).

NHS England has recently funded a study published by the LGBT Foundation (2022) which looked at improving trans and non-binary experiences of maternity services. This report recommends supporting the delivery of personalized care and proactively adopting inclusive language. It also explores trauma-informed perinatal care and targeted outreach to trans birth parents. A focus on the importance of inclusive language is echoed in the updated National Institute for Health and Care Excellence guidance on the use of person-centered language to support respectful, empathetic, and inclusive language for sex, gender and sexual orientation (NICE, 2023). The issue of inclusive language is pertinent as misgendering language provokes harmful feelings of dysphoria (MacDonald et al., 2016). Its significance is also highlighted in a research context with the publication of guidance relating to sex and gender inclusive language (Bamberger & Farrow, 2021), and challenges to assumptions around gender and a lack of inclusive language in human lactation studies (Dinour, 2019). Despite these calls designed to promote health inequality inadequate access to competent healthcare appears to be particularly common for trans and non-binary individuals who are starting a family (Botelle et al., 2021).

Evidence of consideration of trans parents and their babies by maternity and early years services, including health visiting, remains sparse (García-Acosta et al., 2019). Trans parents have reported receiving a lack of information about the birthing and bodyfeeding process and they are more likely to say they did not receive support and encouragement when contacting a midwifery or health visiting team (Bower-Brown & Zadeh, 2021). Indeed, in a case study outlined by Lowik (2015) a transgender parent based in Winnipeg was left to educate himself on bodyfeeding his own child as healthcare professionals assumed that he would bottle feed and supplied him with formula samples. Conversely, some transmasculine people reported pressure to bodyfeed from healthcare professionals and family which led to feelings of anxiety and need to succeed (MacDonald et al., 2016). There are also studies which have reported a refusal of treatments and inappropriate referrals to social services (Hoffkling et al., 2017; Light et al., 2014). Furthermore, the issue of TGNB people nursing their babies is currently socially constructed as vexed, often invisible and problematized (Charter et al., 2018) with particular vitriol directed at trans mothers (Darwin & Greenfield, 2019).

Given that the health benefits of human lactation for babies and gestational parents are globally noted for cisgender women (Ip et al., 2009; Sankar et al., 2015) and the global healthcare policy which recommends a continuation of bodyfeeding for two years and beyond (WHO, 2023), it is important to understand how people within TGNB communities can be supported in their bodyfeeding practices. The evidence presented so far highlights the importance of building a strong relationship centered on respect and person-centered care (Ahmed et al., 2022) with TGNB parents. Furthermore, given the link between improved health outcomes and evidence-based practice (Melnyk & Fineout-Overholt, 2022) it is important that trans and non-binary parents receive evidence-based advice about infant feeding.

Given that TGNB parents experience structural exclusion in healthcare more generally, and specifically within maternity and infant health services, this study aimed to contribute to the recent body of literature exploring the needs of trans and non-binary people during early parenting as a continued effort for addressing health inequity. For this exploratory study, parents were invited to take part if they had personal experience with chestfeeding and/or breastfeeding. This current research includes trans men, trans women, and non-binary parents’ experiences of chestfeeding or breastfeeding their babies, in the UK.

## Method

This paper used a qualitative approach informed by a reflexive thematic analysis (RTA) (Braun & Clarke, 2021) to explore the breastfeeding and chestfeeding experiences of parents within trans and non-binary communities. This approach was chosen as it provides a rigorous, systematic approach to identifying key themes within qualitative data and acknowledges the importance of researcher subjectivity within the process (Braun & Clarke 2021).

The study was initiated and designed by two cisgender researchers, JEJ (a registered SCPHN) and JH (a psychologist). JEJ is a breastfeeding researcher who initiated the study in response to a direct request from trans people to widen her research scope. RW (a registered midwife), who is trans, joined the team after the initial design but before recruitment. PS, a research assistant, also joined the team at this point.

An engagement group comprised of three queer and/or trans people with experience in infant feeding or professional lactation support played an integral role in the study design. Members of the group were contacted *via* trans charities, as well as personal and professional contacts. A concerted effort was made to invite people from diverse backgrounds so that a range of perspectives were brought together. However, most of the members of the research team are white with only one member of the engagement group belonging to an ethnic minority group. This limited the scope and cultural competency and is considered in the findings.

The engagement group consisted of a queer person who is an International Board-Certified Lactation Consultant (IBCLC), doula, LGBTQI + author, and breastfeeding parent (BJW), a trans woman with experience of inducing lactation for breastfeeding (anonymous), a trans-masculine non-binary senior midwife who trained and worked in the NHS across settings (RG). The group met monthly between January and July 2022 to provide feedback on the study design, the analysis and the write-up of the results. All members of the engagement group received payment of £10 amazon evoucher for each meeting they attended. This payment was given in recognition of their time and valuable expertise. As such, the design of the study can be considered participative production and the analysis to be inter-disciplinary and intracommunity.

### Data collection and participant information

Data were collected from 7 participants (outlined in Table 1) who responded to a recruitment call shared by trans organizations through Twitter, Facebook and Instagram. An initial phase of deeper outreach to Black and other minoritized trans parents was unsuccessful in recruiting Black or Southeast Asian parents.

**Table 1.** Summary of participant information.

Pseudonym	Ethnic group	Identity	Personal pronouns
Rhys	White British	Trans man	He/him
Adam	Korean Irish	Trans man	He/him
John	White British	Trans man	He/him
Nick	Participant chose not to specify	Trans man	He/him
Ailish	White British/Irish	Trans woman	She/her
Aumerle	White European	Non-binary	they/them
Rowan	White British	Non-binary	they/them

A decision around sample size was made *in situ* during the project and the volume of data was monitored as it was being collected (Braun & Clarke 2021). In part, the sample size was determined by the practical constraints of working with minority populations in a timescale set by funders. Future research must consider this from the outset.

TGNB parents can choose to feed their baby human milk in multiple ways (Woodstein, 2022). These are physiologically initiated breastfeeding or chestfeeding, medically induced lactation (Goldfarb & Newman, 2015), and the use of human donor milk (Kontopodi et al., 2021). Some parents also chose to nurse their babies while giving them supplementary milk (mixed feeding). Both human and supplementary milk can be given *via* a nursing supplementation system or a bottle. The diversity of feeding choices was reflected in the participants involved in the study.

To protect the identities of participants information about the participants’ age, their children and their location is not provided in the table. To give general insight into demographics the age range of participants was 24–41 years (mean age 33 years). Four participants had one child and three participants had two children. The ages of the children spanned from 24 days to 14.5 years. In terms of geographical location one participant was based in a city in Scotland, one participant was based in a city in the east midlands, three participants were based in city locations in the south of England, and two participants were based in Wales with one participant living a a city and another in a town.

Data were collected using a semi-structured interview schedule that invited the participant to reflect upon their reasons for choosing to try bodyfeeding. Additionally, their experiences of establishing and continuing their feeding practice with recommendations for other parents seeking to make the same decision were explored. All participants received payment of £10 amazon evoucher as a gesture of thanks for their time and insight.

All interviews were dyadic and were conducted online using Microsoft Teams by either JJ or RW. There were no significant differences between the types of information shared with the different researchers. This is noteworthy given the insider status of RW and the outsider status of JJ. The interviews lasted between 33 and 65 min (an average length of 45 min) and were transcribed verbatim (with any identifying information removed or changed) for analysis.

### Ethical considerations

Before taking part in the study all participants received (i) information about the study that outlined what their involvement entailed, how their data would be stored securely and how their identity would be protected and (ii) a copy of the interview schedule. This information allowed the participant to give informed consent ahead of the interview and provided an opportunity for them to select a pseudonym that is used throughout the analysis. After the interview, all participants were de-briefed; as part of this, they were reminded of their right to withdraw and signposted to trans charities that could be contacted if further specialist support was required. The study was approved by the University’s ethics committee (ref ETH 2122-3794).

### Analytic approach and procedure

The analysis was guided by Reflexive Thematic Analysis (Braun & Clarke, 2021). Given the small, exploratory nature of the project, and a desire to learn from trans and nonbinary people about their experiences, themes were identified using an inductive approach. This requires the analyst to consciously put aside preconceptions of what may be important/present in the data based on personal experience or professional interest and allow the participant’s words to guide the analysis as much as possible (Braun & Clarke, 2021). The differing insider and outsider identities of the research team and engagement group worked to support the analytic process and the identification of themes using the 6-step process outlined by Braun and Clarke (2021) outlined in Table 2.

**Table 2.** Stages of the analytic process.

Analytic stage	Process
Familiarisation	RW and JH read through the interview transcripts several times and noted down initial notes.
Generating initial codes	JH and RW coded interviews independently. Disconfirming evidence and contradiction was captured during the coding process to capture the complexity and richness of the data.
Searching for themes	JH and RW met with the wider research team to discuss their initial coding. JH talked through the candidate themes that she had identified and extracts relating to them. RW presented a visual representation of the candidate themes that he had identified. There was a large level of agreement.
Reviewing themes	JH collated extracts together for the themes identified and agreed with RW. This document was sent to members of the engagement group for their feedback.
Naming and defining themes	Themes were named and finalized in line with feedback from the engagement group members. Extracts chosen by the engagement group for each theme were included in the analysis. The analysis was informed by an experiential perspective (Braun & Clarke, 2021c) which provides insight into the unique experiences of the speaker (Clarke & Braun, 2013)
Producing the report	RW and JH developed the analytic commentary and the research team worked together to write up the paper. A draft of the paper was sent to the engagement group for their feedback. The report was finalized in line with this.

## Findings

As outlined in Table 3, the analysis identified three themes which focused on (i) the baby and doing the right thing for them, (ii) the experiences of a changing body and the support desired whilst navigating this, and (iii) the experiences the parents faced with HCPs throughout their feeding journey.

**Table 3.** Summary of themes and subthemes explored in the analysis.

Theme	Subthemes
Baby	*Health benefits* *Bonding* *Comfort and convenience* *Stubbornness*
Body	*Confronting experiences with a changing body* *Physical challenges*
Support in healthcare settings	*Inclusive language* *Feeling represented*

## Baby

The baby was the center of the parents’ experiences and the parents were well informed of the benefits of human milk. This shaped their decisions around their bodyfeeding practice. When discussing their decision to bodyfeed, many of the parents stressed the health benefits for their baby and said this was a strong motivator.

*I believe that it’s the best thing for them, like for immunity boosting and everything. We went for vaccinations, I just want to do the best for her. And I’m a really science-based person (Adam, trans man)*

Here, Adam focuses on doing *the best* for his baby and this is situated within a wider narrative of protection. Human milk is aligned with other measures that play a role in building immunity, such as vaccinations. There is a sense that Adam plays an integral role in putting his daughter’s needs first by ensuring that she receives the best possible care. For other parents, too, an evidence-based approach helped to support the continuation of bodyfeeding.

*I know there’s health benefits, and it’s quite amazing how your body changes to their needs. (John, trans man)*

Within John’s account, there is a feeling of awe for how his body adapts to the infant’s unique needs along the bodyfeeding journey. This implies a continued connection through bodyfeeding and highlights the parental role of meeting the infant’s needs. This interconnection was also reflected in the emotional bond it facilitated.

*I wanted the connection with our baby. I didn’t want to be, you know, the other mummy. And obviously like to some extent I am another mum because you [the baby] lived inside my partner for nine months, nearly. You were early. But to the extent that I could, I wanted that kind of connection and that bonding that now that they’re out. And to try and have as, as equal a division of the labor as is practical given the limitations of biology. (Ailish, trans woman)*

For Ailish, the desire to lactate was linked to developing an emotional connection that was central to developing equity in her parental role. This issue was echoed by other parents who were in relationships and recounted ways they navigated decisions around pregnancy and lactation. By sharing breastfeeding Ailish both deepens the bond she has with her baby and practically supports her partner. Rhys’s recollection of night feeding further explores the demands of chestfeeding and the role that connection played.

*Like the late nights, at three in the morning when it was just me and him awake and you’re just sat there, looking, and it’s just a really nice feeling of connection. Yes, I feel like that’s the most positive thing about chestfeeding. (Rhys, trans man)*

Rhys’s focus on positive feelings and a sense of being at one with his son shifts the emphasis around sleep deprivation, which is often associated with night feeding, toward a tender moment. Here chestfeeding is presented as a deeply rewarding experience. Other parents acknowledged the link between the comfort infants received from chestfeeding.

*It’s like a magic potion. You want the child to sleep? You want the child to stop crying because she fell over? Ooop. You want her to be quiet during a meeting? On the boob (Aumerle, non-binary person)*

Aumerle presents the practical convenience of human milk. Significantly, there is also a focus on sleep; this was important as many parents spoke about the demands of bodyfeeding through the night and the exhaustion this brought about. There is a sense that bodyfeeding is part of the everyday routine which plays an important part of family life. However, there was not always a sense of ease in relation to bodyfeeding as parents recounted tension around a strong determination to bodyfeed and fear of failure.

*I really wanted to do it, for her, and a little bit of like- You know, my body should be capable of it, I’d like to do it, just to see if I can. (Adam, trans man)*

Adam’s account places his daughter at the center of his bodyfeeding choice but there is also a sense of testing his body which negatively impacted his wellbeing.

*It’s also selfishly a bit tied to my self-worth. Or it feels very tied, actually; my husband tells me off for feeling quite worthless when I can’t do it, or when it does drop. (Adam, trans man)*

Adam’s sense of value as a parent is closely tied to milk production and meeting the needs of his infant. This implies that Adam places a huge amount of pressure upon himself to lactate and when this is not possible, he feels *worthless*.

This section of the analysis has highlighted how the parents’ understanding of the benefits of human milk informed their bodyfeeding choice but also introduced a sense of pressure to succeed. The concept of pressure and the physical and emotional demands this placed on the parents is now explored in the theme of the body.

## Body

During bodyfeeding, the changing body was confronting for parents which raised concerns about dysphoria. Consequently, bodyfeeding led them to develop new relationships with their chest area. For the trans men, who not had previously accessed gender-affirming top surgeries, this involved changes in binding. The non-binary participants did not bind—for one participant this was a choice and for the other a medical condition prevented binding. However, seeing a part of their body, which they were not comfortable with, grow presented significant challenges.

*I always felt quite uncomfortable with that part of my body and when I was chestfeeding both times, I had massive touch aversion… So literally couldn’t stand to be touched by anything or anyone. So like my partner, my pets, anything I couldn’t stand it. So yes, it was quite difficult (John, trans man)*

John’s account explores how chestfeeding magnified uncomfortable feelings toward his body which negatively impacted his chestfeeding journey. However, there is a sense of strength and determination as John faced the difficulties and continued through this discomfort. Within his interview, John explored the wider impact that chestfeeding had on his well-being.

*I think the biggest challenge for me was overcoming my own physical issues. Like the sensory aspect, the emotional aspect, the baggage I guess, that that came with. And that was really difficult for me, and I was in quite a dark place with it because I didn’t really want to be doing it. (John, trans man)*

Chestfeeding is presented as an overwhelming experience which manifested difficult emotions. John’s *dark place* evokes a sense of isolation during this extremely difficult time. This experience highlights the need for awareness from HCPs of the issues TGNB people experience to support their well-being whilst lactating. This issue was elaborated on by Adam as he spoke about the issues he encountered when exposing his body to HCPs to receive lactation support.

*I remember that time feeling like a lot of strangers were seeing my body that I don’t like. Yes, a bit raw (Adam, trans man)*

Adam’s use of the word strangers suggests a lack of relationship with his care providers. This amplifies the *raw* feeling of vulnerability when repeatedly showing body parts that he was in the process of coming to terms with. This illustrates the need for sensitivity and person-centered care. When reflecting upon the changes in his body Adam reported conflicting emotions.

*I don’t have that [relaxed and accepting] relationship with my body. I think it’s improved slightly from the experience, having to look at it all the time. Yes, I have a very, very large chest, too, so that’s been unfortunate. It’s tripled since pregnancy. So my relationship with my body has changed. A little bit more positive, I think, but also quite angry at my body. I sort of resent the experience as well. It was nasty to start with, and I’m just looking forward to top surgery. (Adam, trans man)*

The confronting experiences of having to look at his body and acknowledge the changes taking place are presented as a key to initiating a more positive relationship with his body. However, there are also strong negative feelings toward the body and this conveys a complex relationship in which the body is accepted but also rejected. Adam discussed this further concerning dysphoria.

*I was really worried that I’d get quite a bit of gender dysphoria. Funnily enough, it eased for me, because I finally had a use for the stupid things… Suddenly they were useful, they were like a… They suddenly became a tool. It’s a much more relaxed relationship with it now, but at first, it was quite an intense new skill to learn with a body part that I hate. (Adam, trans man)*

Adam highlights the intensity of his bodyfeeding experience and situates this within a narrative of acceptance. Within the extract, his body was conceptualized as a *stupid* body part. This illustrates the challenges when establishing bodyfeeding and the determination required to work through these difficulties. However, seeing his body part in terms of a tool which enabled bodyfeeding was key to a shift toward ease. Rhys also picked up on this issue.

*I’d like, hold my chest as if it was a bottle, which was a really- I don’t know, maybe the disconnect of that, I don’t know whether cis women do that, but yes, it made it feel more like a separate thing. (Rhys, trans man)*

Viewing his chest as a bottle enabled Rhys to manage his feelings of dysphoria. In contrast to Adam, maintaining a sense of disconnection from his body was an essential part of his chestfeeding experience.

Whilst managing feelings of dysphoria the parents also dealt with a range of physical issues associated with bodyfeeding such as thrush, pain, and nipple damage. Typically, these issues were faced at the beginning of the bodyfeeding journey whilst working through the confronting experiences with their body. However, for Aumerle, feelings of discomfort persisted as their bodyfeeding moved past 12 months.

*I think mainly the pain, it was just really, really painful at the beginning and even now sometimes that it gets uncomfortable, especially if she’s ill or something like that and she will only sleep on the boob, and she just hangs there for two hours kind of thing. (Aumerle, non-binary person)*

Aumerle’s experiences mirrored the experiences of others in the sense that discomfort had to be endured and worked through for the good of their child. Indeed, Aumerle recounted how this was reinforced by HCPs who told Aumerle not to ‘worry’ about the pain as their baby was gaining so much weight. Dismissal of parental concerns can damage trust in healthcare, and it is important that TGNB parents feel able to reach out for support.

Whilst parents included in the study shared many universal challenges in infant bodyfeeding, there were also issues which were specific to trans parents. For example, Ailish raised concerns about the volume of ‘off label’ medication required to breastfeed beyond infancy.

*I’d like to continue being able to feed at least through the first year. I’m not sure after the first year because it involves a truly spectacular number of tablets a day and the prospect of doing that for years and years doesn’t necessarily appeal… But I would intend and aspire to keep going with as much as I can for at least the next 12 months. (Ailish, trans woman)*

Ailish contrasts her breastfeeding aspirations with the demands of taking the medication required to induce lactation over a prolonged period. She also raised concerns about the side effects of using the medication long-term. As such Ailish’s breastfeeding journey is mediated by careful consideration of the demands it places on her body. For the trans men included in the research study, testosterone became a point of contention with their HCPs.

*She [specialist doctor at a gender clinic] said that she wouldn’t put me back on testosterone if I was still chest feeding. Well I know that people have chest fed and been on testosterone, apparently it’s fine because apparently the molecules like separate, it’s bigger than the molecule to go into the chest milk. I don’t know. So, it shouldn’t go through. But the only studies that have been done have been done on cis woman taking an oral version of testosterone, not like a trans man where the level would be different, I think.” (Rhys, trans man)*

Rhys recounts a disparity between the advice given by his healthcare provider, the research evidence and the knowledge shared within his community. The conflicting evidence seems to result in a sense of uncertainty and exasperation for Rhys ‘*I don’t know’* and pausing testosterone was another sacrifice that he made for the good of his child. Furthermore, there is no suggestion that Rhys was able to discuss his feelings and concerns as traditional, unidirectional interaction between doctor and patient is evidenced. The importance of open, patient centered communication was expanded upon by Adam.

*Yes, they [healthcare providers] really didn’t want to put me on it, because they’re like, “Well, it’s unknown, it might harm your baby,” they really made me feel awful. Like, a few breakdowns over that. Just crying, thinking if I was doing the right thing, but I’ve known somebody who did it 12 years ago on T [testosterone]. Their children are fine, all three of them. Because obviously, everyone communicates in the Facebook groups, really, but. Not in a group antivax mummy kind of way, but in a here’s the evidence. These three studies. (Adam, trans man)*

Adam eventually won what he termed *the battle* to continue testosterone whilst bodyfeeding. However, this came at great emotional cost and Adam turned to his community for support and evidence to help inform his decision. This was common within the interviews as TGNB parents regularly turned to their community, friends and family for emotional support and medical information that they felt was lacking in healthcare settings.

As already evidenced earlier in Adam’s account top surgery was something that he was looking forward to once his bodyfeeding journey reached an end. This issue was also addressed by Nick.

*I think wouldn’t it be great if we had a world where like the surgeons who are the best at top surgery could give their patients around retaining the ability to chestfeed because that’s absolutely possible. (Nick, trans man)*

Nick calls for better communication from HCPs around providing information and options around top surgery. Such support would help to address some of the physical issues faced by trans men as they chestfeed.

This theme has examined the range of issues that parents faced as they moved through their bodyfeeding journey. The analysis ends by examining how HCPs can support trans and non-binary parents.

## Support in healthcare settings

Many of the parents spoke about the practical support they received from HCPs for developing a good latch. Whilst this was appreciated, there was also a sense that emotional support was sporadic depending on services available in the area the parent lived. Further to this, parents expressed the need for more accessible, specialized support concerning bodyfeeding.

*I talked to the local lactation consultant that I was having like quite significant feelings of nausea and upset while pumping, which had been going on for months and she basically said “aha, this is absolutely classic D-MER (dysphoric milk ejection)” which I wish I’d known before because I’d have known what was going on… I wish I’d known about that sooner because I think knowing that’s what’s going on, there was quite a lot of angst in, or pumping makes me sad, do I not want to do this? (Ailish, trans woman)*

As already evidenced, parents dealt with the physical and emotional issues alone and viewed these issues as something they had to battle through for the good of their infant. Ailish’s account illustrates this further and demonstrates the need for specialist support early on. Such support would have helped to address the *angst* she felt.

The importance of inclusive language for HCPs was also apparent. Within the interviews, parents recounted being misgendered and HCPs using the default term of mum. Parents, and in some cases their children, needed to address gendered assumptions expressed in healthcare settings.

*I think my main thing would be something about more gender-inclusive language, that it is a very big wall of mummies, the kind of thing that hits you language-wise (Aumerle, non-binary person)*

Aumerle creates a powerful image which highlights the importance of language for inclusivity. Other parents shared the feeling of being an outsider and hitting a seemingly impenetrable wall in terms of gendered language which rendered them invisible. There was an acknowledgement that addressing gender may be challenging for some, for fear of getting it wrong or causing offense, but the importance of asking questions was stressed.

*Trans people that- They want to be seen. And they want you to ask the questions. They just want to be seen… yes. A simple question of “What’s your pronouns?” Or “What’s baby going to call you?” (John, trans man)*

John highlights simple questions that can be used to open discussion around gender to facilitate person-centered care. Practical solutions which removed the need for TGNB people to engage in multiple conversations around pronouns were also shared.

*There could be a way of signifying it’s a trans man in the room, they go by dad. Because even if someone has to- Like cis women parents, maybe one of them doesn’t go by mum, so maybe something that can say these are the terms that they want to use. (Rhys, trans man)*

The use of language was also very important when it came to lactation support, even when sensitivity was shown toward pronouns.

*I was like, “Oh, I’m trans, by the way.” Although, I didn’t really have to remind them, because my care providers here were amazing. They–told everyone ahead of time, they were like, “this is, you know, the language that Adam uses,” but- Everyone was good at using it for me, but then when they were talking broadly, they were like “breasts,” (Adam, trans man)*

Adam highlights the need for consistency around language and person-centered care. There is a sense of appreciation for the effort his HCPs put in to ensure that all the practitioners involved in his care were fully briefed as this removed some of the mental load for him. However, when it came to discussing lactation in general, the same level of awareness was not present. As previously outlined, the parents faced complex issues relating to dysphoria as they moved through their bodyfeeding journey and so awareness of language is important when offering lactation support.

*Yes, so I prefer the term chestfeeding for myself obviously because I’ve had top surgery now, but even then I didn’t resonate with the idea of having breasts and it’s quite an uncomfortable word, like a dysphoria triggering word, because even before it was easier- Because I used to wear a binder so it would just look like I had a flat chest. But the term ‘breastfeeding’ just feels a bit weird for me, like it creates the thing, in my stomach, of dysphoria which I’ve felt in the past more. But it’s starting to go away now, which is good. But, yes, especially in the early stages of transition, I’d get a tight feeling, like thinking about being called a woman and stuff like that. It kind of gives me a similar sensation of discomfort (Rhys, trans man)*

Rhys’s narrative illustrates the power of language and the physical and emotional impact it had upon him. The use of the word *breasts* is highly triggering as it brings about feelings of regression concerning the dysphoria that he has worked through during his transition. As such it strips him of his progress made in managing the feelings of *discomfort*. Significantly, Rhy presents the term chestfeeding as the language that he finds most affirming, thereby highlighting the need for conversations about language options between healthcare providers and their patients. Indeed, within the interviews, there was diversity in terminology.

*I like body feeding, as well. That feels very powerful. Sort of like a reminder, you’re doing it with your body. From the body. (Adam, trans man)*

Adam identified how the most affirming terminology was that which evoked feelings of empowerment and within his interview, he also explored how representation in lactation support was needed.

*When I was in the hospital, having just given birth, the person- Like, lactation expert came around, showed me this book of naked women chestfeeding in different positions, hair down, completely feminine, husband in view in the background, that was the photography. And then, she was just like, “Ooh, I’m sorry, this is all we have to show you some positions and stuff. We’ve got to update it.” And it just- That- What I want from healthcare professionals is, update it then. Get some models in that are trans. Take some photoshoots with a baby. These people exist. That would be fantastic to have a book. (Adam, trans man)*

Within Adam’s healthcare setting, the cisheteronormative assumptions present in the bodyfeeding support is problematized by the lactation expert. There is a shared feeling that more representation is needed within the images presented to enable trans people to be seen and feel included. Adam went on to explore the wider implications of representation.

*I just think if the language was used around these kind of mummies that are in my group, that are pretty mean, that if they were shown the same picture group, with like- Men in it, feeding, with trans people in it, feeding- People that weren’t white, feeding- Maybe they would speak to me a bit more in group (Adam, trans man)*

Wider representation is presented as being the key to educating all patients about issues of diversity and promoting acceptance.

## Conclusion

These findings have highlighted experiences of TGNB parents, which in some ways mirror universal challenges concerning infant feeding. These parents were knowledgeable of the benefits of bodyfeeding their babies human milk and this was a strong motivator to put the needs of their babies first. Despite not facing pressure from the healthcare providers as previously reported by MacDonald et al. (2016) the participants’ choice to bodyfeed was firmly influenced by the wider rhetoric of *breast is best*. At times, bodyfeeding was expressed as a positive experience that enabled the parents to use their bodies to nurture and connect with their child. For Ailish in particular breastfeeding was a gender affirming and empowering experience which enabled her to deepen her relationship with her wife and create a unique bond with her baby.

At other times, the parents experienced psychological challenges which left them feeling unsure of their abilities, disconnected from their bodies, isolated or with a lack of self-worth. Bodyfeeding also triggered distressing feelings of dysphoria. Echoing findings presented by MacDonald (2016) the new-found utility of the parent’s chest area was a coping strategy used by some participants to manage their distress. However, the withdrawal of gender affirming testosterone treatment and a lack of advice around top surgery meant that the trans men encountered limited medical support in relation to the dysphoria they felt. This aligns with wider literature which highlights the multiple social and personal challenges during childbearing parenting (Botelle et al., 2021; Falck et al., 2021; Winter et al., 2016) and refusal of treatment (Hoffkling et al., 2017; Light et al., 2014). It also draws attention to the importance of open comminication in relation to top surgery (MacDonald, 2016). As evidenced in the analysis top surgery was postponed by one participant so that they were able to bodyfeed. This echoes the views of trans youth around the need to start a family before transition (Lowik et al., 2023). Consequently, HCPs need to provide better communication and resources concerning options for top surgery and bodyfeeding to facilitate trans people in making informed choices around reproduction, infant feeding and transition.

Whilst the determination to bodyfeed and the persistence of the parents is to be acknowledged, it raises questions around the pressures at the parents faced. Within the wider context of the transnormative reproductive subject ‘there is a social script for what it means to be transgender and the types of experience that are recongized or acepted by society’ (Lampe et al., 2019, p. 1). In line with this it can be argued that the pressure to bodyfeed despite the personal distress it caused for some can be aligned with the pressure to be ‘just as normal as cisgender parents’ (Lampe et al., 2019, p. 13). By conforming to cisnormativity trans people are allowed wider social acceptance (Lampe et al., 2019) but as demonstrated in this analysis it came at a personal cost. In light of the incongruance and distress felt by some participants a wider discussion around feeding choices may be required for TGNB communities. Whilst it is important to highlight the benefits of human milk other forms of infant feeding should also be discussed to help facilitate affirmative feeding choices.

A cisheteronormative environment was relevant in healthcare settings has specialist services were sporadically available and this left the TGNB parents feeling like outsiders. In line with previous literature this is significant as it created a barrier to healthcare services for TGNB people (Norris & Borneskog, 2022). The experiences shared by the parents further reinforced the importance of using gender affirming language in terms for personal pronouns reported by MacDonald (2016). Further to this, use of gender affirming language also needs to be consistently extended to the chest area when HCPs are providing lactation support. To avoid triggering dysphoria HCPs need to establish the parent’s preferred terms for lactation support (breast, chest or body feeding for example). Once these preferred terms and established consistency is required across every encounter. Furthermore, empathy and respect are important when supporting the physical aspects of successful bodyfeeding such as latch. When supporting TGNB parents HCPs need to be aware of the sensitivity and vulnerability and not assume that parents are happy to expose their chest area for lactation support. Open conversation and awareness provide the cues that MacDonald (2019) argued are necessary for a gender affirming environment. This, in turn, helps to establish trust and help address barriers to healthcare that MacLean (2021) had identified as issues that have resulted in the further marginalization of TGNB people.

The need for a person-centered approach which demonstrates empathy and respect (Ahmed et al., 2022) was also highlighted in the need for gender inclusivity within infant bodyfeeding resources. This indicates that whilst recent LGBT Foundation (2022) for NHS England and the updated NICE (2023) guidance on inclusive language good are starting points more can be done in terms of representation. TGNB parents want to be seen and have their experiences recognized in so that resources for new parents are more inclusive and diversity is celebrated. Finally, there is a need for HCPs to have the support needed to share evidence-based advice with trans and non-binary parents as this improves health outcomes (Melnyk & Fineout-Overholt, 2022). As evidenced in the analysis the advice around testosterone and body bodyfeeding given by HCPs was inconsistent and at times contradictory to other evidence. This led to a *breakdown* for one participant and withdrawal of treatment for another. In both cases there was a sense that there was a lack of trust with the HCP and the participants turned to their community for support, information and reassurance. This indicates that healthcare services need to work more closely with trans people and create easily accessible resources that can be utilized by HCPs.

## Limitation

Research on trans and non-binary individuals has predominantly focused on white, middle-class, and non-disabled populations (Vincent, 2018), and this is also true of research on trans and non-binary parents. An intersectional framework, where multiple axes of identity are considered in the analysis, may be most useful for future research which explores the experiences of trans and non-binary parents (Hafford‐Letchfield et al., 2019).

## Notes

1 The inclusive term body feeding is used in the paper to describe human lactation. Breastfeeding and chest feeding are used as terms when the specific preference of the speaker is known.

2 This term is used by Norris and Borneskog (2022) and is therefore cited here. However, the authors would like to acknowledge the negative, ableist connotations of equating blindness or vision loss with ignorance or lack of knowledge
